# Pix2Pix-Based Monocular Depth Estimation for Drones with Optical Flow on AirSim

**DOI:** 10.3390/s22062097

**Published:** 2022-03-08

**Authors:** Tomoyasu Shimada, Hiroki Nishikawa, Xiangbo Kong, Hiroyuki Tomiyama

**Affiliations:** 1 Graduate School of Science and Engineering, Ritsumeikan University, Kusatsu 525-8577, Japan; ri0080xs@ed.ritsumei.ac.jp (T.S.); hiroki.nishikawa@tomiyama-lab.org (H.N.); ht@fc.ritsumei.ac.jp (H.T.); 2 Japan Society for the Promotion of Science, Tokyo 102-0083, Japan

**Keywords:** depth estimation, optical flow, AirSim

## Abstract

In this work, we propose a method for estimating depth for an image of a monocular camera in order to avoid a collision for the autonomous flight of a drone. The highest flight speed of a drone is generally approximate 22.2 m/s, and long-distant depth information is crucial for autonomous flights since if the long-distance information is not available, the drone flying at high speeds is prone to collisions. However, long-range, measurable depth cameras are too heavy to be equipped on a drone. This work applies Pix2Pix, which is a kind of Conditional Generative Adversarial Nets (CGAN). Pix2Pix generates depth images from a monocular camera. Additionally, this work applies optical flow to enhance the accuracy of depth estimation. In this work, we propose a highly accurate depth estimation method that effectively embeds an optical flow map into a monocular image. The models are trained with taking advantage of AirSim, which is one of the flight simulators. AirSim can take both monocular and depth images over a hundred meter in the virtual environment, and our model generates a depth image that provides the long-distance information than images captured by a common depth camera. We evaluate accuracy and error of our proposed method using test images in AirSim. In addition, the proposed method is utilized for flight simulation to evaluate the effectiveness to collision avoidance. As a result, our proposed method is higher accuracy and lower error than a state of work. Moreover, our proposed method is lower collision than a state of work.

## 1. Introduction

In recent years, small drones have been more popular than ever from the perspective of flexibility, low power consumption, and reasonable prices. In addition, the drones are expected to play a variety of roles to take advantage of their convenience. The roles include infrastructure inspection, package delivery, and mobile surveillance cameras. Unlike manned vehicles such as cars and airliners, unmanned drones do not need to be controlled by a person and autonomous flights are becoming practical. In terms of autonomous flights of drones, collision avoidance has been indispensable and regarded as one of the crucial issues. Typically, conventional solutions have employed distance sensors. For instance, Light Detection and Ranging (LiDAR) which can detect long distances are employed [1,2]. Depth cameras or stereo cameras are also employed to perceive distance [3,4,5,6]. However, such sensors with high performance are usually heavy, costly, and power-consuming to equip on a small drone. In contrast, low performance depth sensors can hardly have long-distance vision with high accuracy and would rather increase risk of collisions with objects.

Many kinds of research for autonomous flight of drones have assumed that monocular cameras are often used to detect and recognize objects around the drones [7,8]. Single monocular camera-based depth estimation is also actively researched [9,10,11,12]. However, monocular cameras are not useful at night in terms of their visibility. Instead, infrared cameras are employed to improve the visibility at night, but they do not include depth information, which means the distances between a drone and objects, as well as monocular images. In the literature, depth images have been more important than ever to measure how far the objects are placed from the drone. A lightweight depth camera, which is small enough to be mounted on a drone, can measure a distance up to only 10 m. In addition, unfortunately, high-performance depth cameras are often expensive and too heavy to be equip on a drone. Therefore, depth estimation technology from an image by monocular cameras has been extensively investigated.

The contributions of this paper are threefold as follows:

- This is the first paper to generate a depth image from a monocular image with optical flow for collision avoidance of drone flight.
- We verify that our proposed method can estimate high-quality depth images in real-time, and demonstrates that a drone can successfully fly avoiding objects in a flight simulator.
- In addition, our method is superior to previous method of depth estimation on accuracy and collision avoidance.

The rest of this paper is organized as follows. Related work of drone autonomous flight method and depth estimation method is introduced in Section 2. Section 3 describes the overview of AirSim. Section 4 shows a proposed method to estimate depth with optical flow. Section 5 shows the experimental results and Section 6 concludes this paper.

## 2. Related Work

There has been a great deal of work related to autonomous drone flying for several decades. Much of work has been focused on safe flight, which is particularly necessary to prevent collision with objects. These studies include obstacle avoidance based on ultrasonic, radar, and image processing [13]. Ultrasonic-based methods perform in real-time but the maximum range is short [14,15]. Radar-based methods perform well in obstacle detection. However, radar is not a good choice for small Unmanned Aerial Vehicles (UAVs) due to its weight [16]. Vision-based methods include obstacle avoidance methods based on LiDAR images, Time of Flight (ToF) images, binocular images, or monocular images. In [1,2,17], the authors used LiDAR for collision avoidance of a drone. However, the installation of many powerful sensors or high performance sensors results in an increase in the weight of a drone, which leads to an increase in energy consumption. Given that the drone’s flight is limited by the battery capacity, it is difficult for the drone to fly long distances with a large number of sensors.

In order to tackle this issue, the approaches of [3,4,6] proposed collision avoidance techniques using a light and small depth camera and a stereo camera. The presented methods enable a drone to avoid obstacles on-the-fly by determining an optimum flight direction using depth images. The work in [4], which is inspired by [3,6], proposed an algorithm for collision avoidance by dividing an image from a depth camera into five sections and selects a section so that the section is the most distant object among them. However, depth cameras with reasonable price and relatively low weight such as Kinect released by Microsoft can hardly be mounted on a drone since they can measure only within 10 m [18]. In the context, depth estimation from a monocular camera, which can overlook farther than a depth camera, has been attractive.

In the studies [9,10], the authors presented the methods of depth estimation using Support Vector Machine (SVM). The systems divide an image from a drone into patches, represent each patch using a set of manually created features, and estimate the depth of each patch using a pre-trained SVM classifier. However, the accuracy of their systems is not high as a result of handheld training data. In [19,20,21,22], these methods are based on Convolutional Neural Networks (CNN). The CNN-based methods are more accurate than the SVM method in [9,10], but the accuracy is still not sufficient enough to realize secure flight without collisions. In [11,23], the authors proposed methods to preprocess segmenting images before depth estimation using a monocular camera. This method improves the accuracy of depth estimation. On the other hand, the computational workload largely increases, and the method is not suitable for real-time processing in terms of the performance since the methods [11,23] need segmentation as pre-process. In addition, the methods [11,19,20,21,22,23] use public datasets [24,25] to estimate depth. As the latest work, AdaBins based on a transformer has been proposed [26]. However, the data that the works have used for training and testing in [24,25] are unsuitable for drones view since the data in [24] is suitable for ground vehicles and the data in [25] is oriented to indoor environments. In [12], the authors collected data from drone views in a drone flight simulator and presented a method to generate depth images using Pix2Pix [27] from a monocular image.

Drones are further smaller than general vehicles, and the processing capability of drones is comparatively lower than that of cars since the large computer cannot be equipped on the drone. Most of recent technologies based on image processing have exhausted computational resources due to the development of deep neural networks (DNNs), and the technologies are seemingly not suitable to the system in a small device. However, many embedded systems are oriented to the Internet of things (IoT), and the computation with data transmission to an on-land computer has been enabling to distribute the computational workloads. Although this fashion has spurred the development of depth estimation technologies using a monocular camera, there is little work that focuses on depth estimation for a small drone. Drones are required to fly without colliding objects but the weight of a camera that can be carried by a drone is severely limited, and high performance but heavy cameras cannot be carried.

In this paper, we propose a new depth estimation method for autonomous flight of a drone. Our proposed method can estimate long distance using a monocular image with optical flow. In addition, our model for the estimation is based on conditional generative adversarial networks (CGAN) [28], and the training dataset is collected from AirSim [29], which is known as the virtual flight environment of a drone.

## 3. AirSim

This section describes AriSim [29], which we employ in this work. AirSim is a kind of flight simulator that uses a virtual environment called Unreal Engine 4. This simulator faithfully reproduces the reality in visual information and physics.

**Figure 1:**
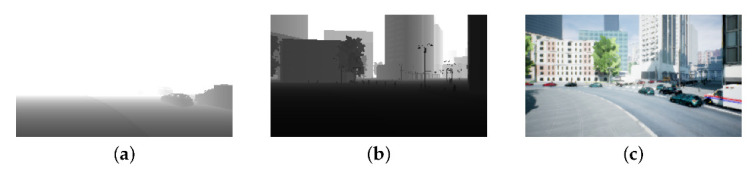
AirSim views: (**a**) Depth map (up to 10 m), (**b**) Depth map (up to 200 m), (**c**) Monocular (RGB) image of (**b**).

In addition, AirSim can acquire mesh information from Unreal Engine 4. The available information includes location, temperature, and images. The obtained images include RGB, segmentation, infrared, and depth images. The depth image in AirSim can exactly measure up to 200 m. Therefore, the AirSim environment enables to obtain and label both RGB and accurate depth images at the same time to create training dataset. Figure 1a shows a depth image up to 10 m taken by a real depth camera that can be installed on real small drones in the AirSim environment. Figure 1b shows a depth image up to 200 m obtained from a depth camera in AirSim. Figure 1c shows monocular image taken by monocular camera in AirSim. As shown in Figure 1, in monocular image and depth image up to 200 m, can detect objects, however depth image up to 10 m cannot detect that. Therefore, the more distance can be measured, the larger the benefit of using depth images for autonomous flight. However, to obtain a 200 m deep image with a real drone, it is necessary to install a camera of great depth, which is unrealistic. Therefore we propose a method for estimating the depth image obtained by AirSim based on a monocular image.

## 4. A Pix2Pix-Based Monocular Depth Estimation with Optical Flow

**Figure 2:**
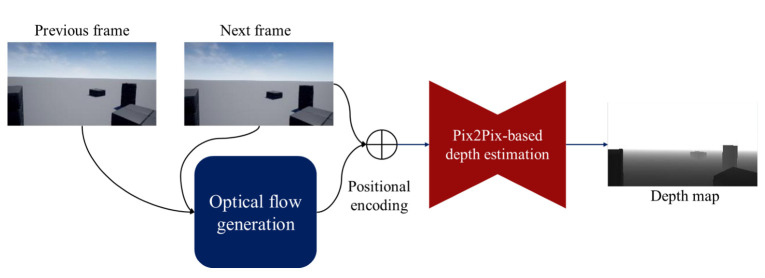
System overview of the proposed method.

This section describes a proposed depth estimation method, which is based on Pix2Pix [27]. Figure 2 shows the system overview of our proposed method. Here, we briefly address our proposed method. The proposed method consists of three parts: The first part generates an optical flow map from two adjacent frames. Second, we combine the generated optical flow map with a monocular image. Finally, the combined image is input into a Pix2Pix-based depth estimator to create a depth image. In the following, we detail each part of the proposed method.

### 4.1. Optical Flow Map Generation

We employ classical methods to generate a optical flow map based on Lucas-Kanade [30] and Farnebäck method [31]. First, we describe the image feature representation and its spatio-temporal analysis in Lucas-Kanade method. Lucas-Kanade method assumes that the deformation of an object between two adjacent frames is slight and that a point *x* on image $f_{t}$ at time *t* has moved by $v_{t}$ on image $f_{t}+1$ at time t+1.

(1) $$\begin{array}{c} \epsilon \left(v_{t}\right)=\sum_{d\in B}{\left(f_{t+1}\left(x+v_{t}+d\right)-f_{t}\left(x+d\right)\right)}^{2} \end{array}$$

Here, *B* represents a certain rectangular window region centered at point *x*, and *d* is a parameter that represents an arbitrary position in the window region. It is the sum of the squares of the luminance differences between the corresponding points in the frame area before and after the movement of point *x*, where $v_{t}$ is the movement from time *t* to t+1. For example, if all the points in the frame region have the same luminance and have moved in the same direction by the same amount, then Equation (1) becomes 0. Approximating the right-hand side of Equation (1) with a first-order Taylor expansion yields the following Equation (2).

(2) $$\begin{array}{c} \epsilon \left(v_{t}\right)=\sum_{d\in B}{\left(\nabla f_{t}^{T}\left(v_{t}\right)-\dot{f_{t}}\right)}^{2}\;\left(\nabla f=\frac{\partial f}{\partial x},\;\dot{f_{t}}=\partial f_{t}=\partial t\right) \end{array}$$

Here, $\nabla f_{t}$ represents the horizontal and vertical difference values of the image $f_{t}$ at point *x*. $f_{t}$ represents the time difference value between adjacent frames at point *x*. Lucas-Kanade method [30] is used to find the displacement *v* at each position *x* that minimizes the sum of the squared luminance differences. By differentiating Equation (2) by $v_{t}$ and setting it to 0, the optimal travel distance $v_{t}$ is obtained as follows:

(3) $$\begin{array}{c} v_{t}=-G^{-1}b \end{array}$$

(4) $$\begin{array}{c} G=\sum_{d\in B}\nabla f_{t}^{T}{\left(\nabla f_{t}\right)}^{T} \end{array}$$

(5) $$\begin{array}{c} b=\sum_{d\in B}\dot{f_{t}}\nabla f_{t} \end{array}$$

Since the amount of movement at each point is not independent, the following iterative process is performed until $v_{t}^{k+1}$ does not change at all points *x*, and the amount of movement is determined.

(6) $$\begin{array}{c} v_{t}^{k+1}\left(x\right)=v_{t}^{k}\left(x\right)-Gb \end{array}$$

On the other hand, Farnebäck method [31] approximates the luminance value of each pixel with a second-order polynomial, and estimates the amount of movement with high accuracy by comparing the coefficients between frames. Let $f_{t}\left(x\right)\in \left[0,1\right]$ denote the luminance value of coordinate *x* at time *t*. The luminance values in the neighborhood of *x* are expressed as second-order polynomials, and the coefficients are optimized by the weighted least-squares method in Equation (7).

(7) $$\begin{array}{c} \hat{f_{t}}\left(x\right)=x^{T}A_{t}x+b_{t}^{T}x+C_{t} \end{array}$$

$A_{t}$, $b_{t}$, $c_{t}$ are a (2,2) symmetric matrix, a (2,1) column vector, and a scalar, respectively. Let $v_{t}$ denote the movement of point *x* at time *t* until time t+1. From $\hat{f_{t}}\left(x\right)=\hat{f_{t+1}}=\left(x+v_{t}\right)$, the movement $v_{t}$ can be estimated as Equation (8).

(8) $$\begin{array}{c} v_{t}=-\frac{1}{2}A_{t}^{-1}\left(b_{t+1}-b_{t}\right) \end{array}$$

In order to obtain a stable solution, Farnebäck method approximates the coefficient $A_{t}$ as follows Equation (9).

(9) $$\begin{array}{c} \hat{A_{t}}=\frac{A_{t}+A_{t+1}}{2} \end{array}$$

Then, using $\hat{A_{t}}$ instead of $A_{t}$ in Equation (8), we obtain Equation (10).

(10) $$\begin{array}{c} \hat{A_{t}}v_{t}=\Delta b_{t} \end{array}$$

Equation (10) holds for all points *x*. Farnebäck method also considers the neighborhood around a point *x*, and introduces the following energy function.

(11) $$\begin{array}{c} \epsilon \left(v_{t}\right)=\sum_{d\in B}w\left(d\right)\left|\right|\hat{A_{t}}\left(x+d\right)v_{t}\left(x\right)-\Delta b_{t}\left(x+d\right){\left|\right|}^{2} \end{array}$$

To minimize this energy, determine the ideal displacement $v_{t}\left(x\right)$ at point *x* is determined to minimize this energy. Farnebäck method is the same as Lucas-Kanade method. Farnebäck method is similar to Lucas-Kanade method, and is obtained by differentiating Equation (11) by $v_{t}\left(x\right)$ in the following:

(12) $$\begin{array}{c} v_{t}\left(x\right)=G^{-1}h \end{array}$$

(13) $$\begin{array}{c} G=\sum_{d\in B}w\left(d\right)\hat{A_{t}^{T}}\left(x+d\right)\hat{A_{t}}\left(x+d\right) \end{array}$$

(14) $$\begin{array}{c} h=\sum_{d\in B}w\left(d\right)||\hat{A_{t}^{T}}\left(x+d\right)\Delta b_{t}\left(x+d\right){\left|\right|}^{2} \end{array}$$

**Figure 3:**
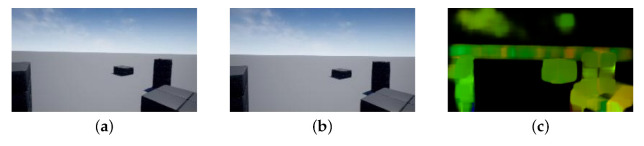
Optical flow map generated from inputs and outputs: (**a**) Previous frame, (**b**) Next frame, (**c**) Optical flow map.

The actual displacement is estimated by iterative operation based on the above equation as in Lucas-Kanade method. Farnebäck method can obtain the concentration gradient stably by approximating the local image surface with a quadratic surface. In general, Farnebäck method provides more accurate tracking than Lucas-Kanade method, although the computational cost increases. Figure 3 shows inputs and an optical flow map using Farnebäck method [31]. As shown in Figure 3, the luminance value of near objects in the optical flow map is high. This figure indicates that relative motion of the objects near a drone becomes large, while that of the objects far away from a drone becomes small. Hereby, we obtain the optical flow map in this way.

### 4.2. Pix2Pix

**Figure 4:**
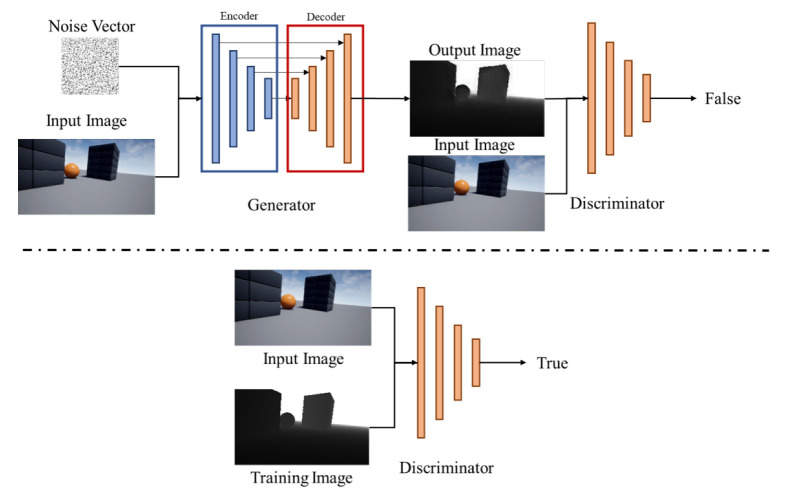
CGAN Network of Pix2Pix Architecture.

**Figure 5:**
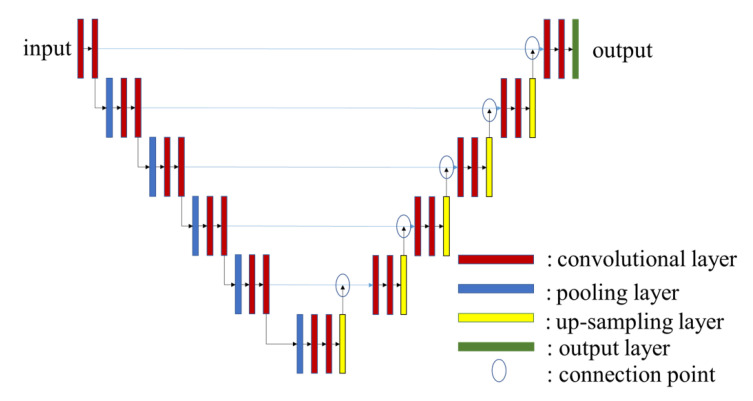
U-Net Architecture [32].

In this work, our proposed method is based on Pix2Pix to generate a depth image from an monocular image [27]. Pix2Pix is well known method simlilar to CGAN [28]. Figure 4 is the overview of Pix2Pix, which represents the broad structure of the CGAN model. CGAN is typically split into two networks such as a generator and a discriminator. The generator learns to prevent the generated image from being detected by the discriminator as the generated one. The discriminator learns not to misidentify the training data and the generated data, and finally the generator is improved by the discriminator and can generates an image similar to the training data. The generator uses U-Net [32], which can extract local features and recover location information, and we show the concept of U-Net in Figure 5. The convolutional layer can extract local features as the layers get deeper. However, at the same time, the location information becomes ambiguous. Therefore, as shown in Figure 5, by sending the location information to the decoder side of the same layer, it is possible to extract local features and recover the location information.

The objective of the CGAN that we have employed is as shown in the following equation, which is referred to [27].

(15) $$\begin{array}{cc} L_{CGAN}\left(G,D\right)=E_{i,gt} & [\log D(i,gt)]+\\ \; & E_{i,n}[\log\left(1-D\left(i,G\left(i,n\right)\right)\right] \end{array}$$

Here, *i* is an input image and gt is ground truth. D(i,gt) is the probability of judging the training data as training data, and D(i,G(i,n)) is the probability of judging the generated image as training data. Let G(i,n) denote the generated image and *n* be a noise vector. The noise vector *n* is not necessary, but if training without *n* input, it results in poor flourishing performance. Therefore, this paper assumes to require the input of the noise vector. The discriminator tries to maximize this objective, while the generator tries to minimize it, and the generator needs to generate images that not only fool the discriminator but also come closer to the ground truth. For this purpose, it is effective to add the following *L*1 norm to the objective of CGAN.

(16) $$\begin{array}{cc} L_{L1}\left(G\right)=E_{i,gt,n} & \left[||y-G\left(i,n\right)|{|}_{1}\right] \end{array}$$

*L*1 norm-based image generation captures the whole image but the blurred details remain a problem. On the other hand, although CGAN-based image generation cannot capture the whole image, it is able to capture the details. By combining these two methods, an image with high accuracy can be generated. Therefore, the objective of Pix2Pix is as follows. *w* is the weight of *L*1 norm. This parameter can be set during training.

(17) $$\begin{array}{c} G^{*}=arg\;\min_{G}\;\max_{D}\;L_{CGAN}\left(G,D\right)+wL_{L1}\left(G\right) \end{array}$$

### 4.3. Depth Estimation Method

**Figure 6:**
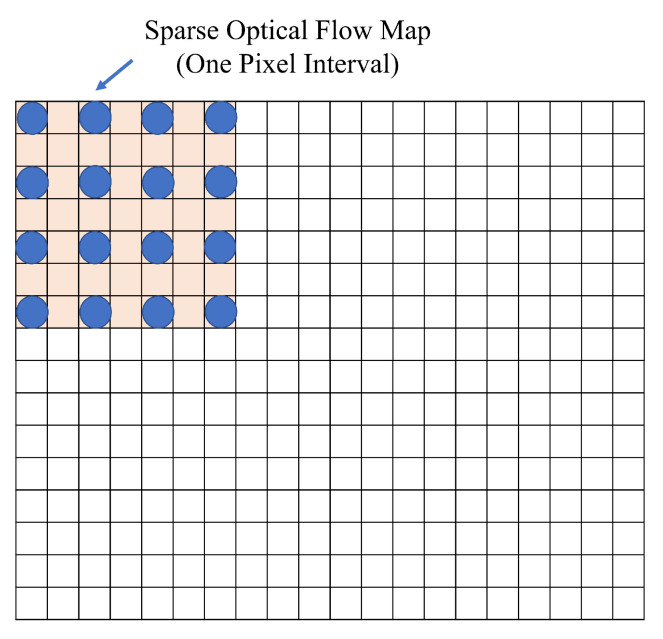
A concept of embedding optical flow map into an RGB image.

In order to effectively use the optical flow map and RGB image for depth estimation, we need to combine them. The concept of our proposed method is based on the atrous convolution in [33], and we exploit a heat map from the luminance values of the optical flow map and embed it into the RGB image. The heat-map is embedded at a certain number of intervals such that the features in the original RGB image is not lost. Figure 6 shows an example that a optical flow map is embedded into an RGB image. The figure utilizes a sparse optical flow map with a single pixel interval. Each pixel is embedded into the original RGB image.

**Figure 7:**
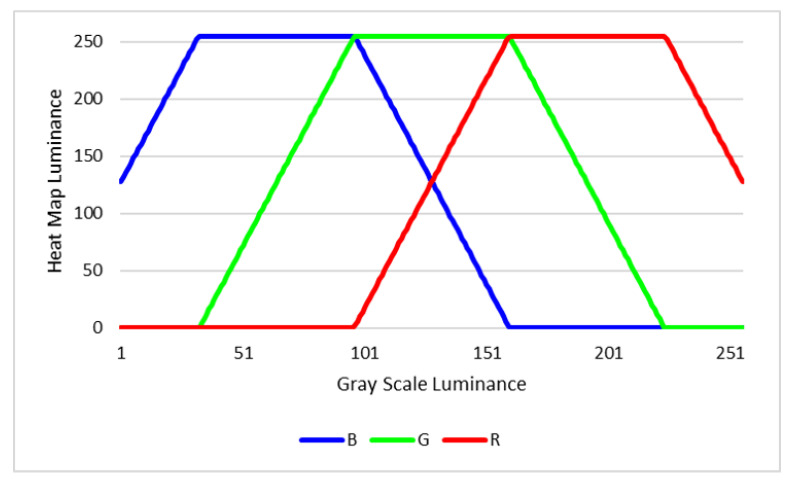
Heat map luminance towards gray scale luminance.

The luminance of red, green, and blue towards gray scale luminance is corresponded as shown in Figure 7.

**Figure 8:**
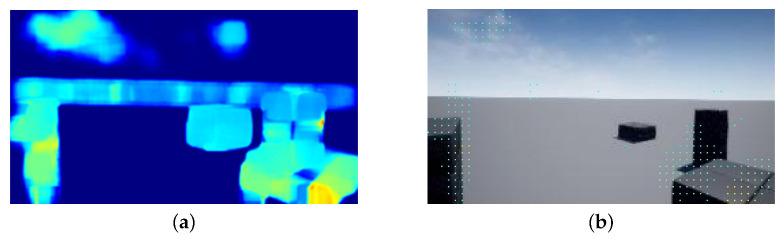
An example of embedding optical flow map into an RGB image: (**a**) Heat-map of optical flow map, (**b**) RGB image embedded with a sparse optical flow map.

In this work, we embed part of the pixel information of the heat map image into an RGB image to generate a depth image with a single-channel input, shown in Figure 8. This embedding method is expressed in Equation (18).

(18) $$\begin{array}{c} E_{\left(n,m\right)}=\left\{\begin{array}{c} M_{\left(n,m\right)}\;\left(n\;\mathrm{mod}\;i\neq 0\;\cup \;m\;\mathrm{mod}\;i\neq 0\;\cup \;O_{\left(n,m\right)}=0\right)\\ O_{\left(n,m\right)}\;\left(n\;\mathrm{mod}\;i=0\;\cap \;m\;\mathrm{mod}\;i=0\;\cap \;O_{\left(n,m\right)}\neq 0\right) \end{array}\right. \end{array}$$

$E_{\left(n,m\right)}$ represents the pixel value of the optical flow map at the pixel position of (n,m) embedded in the monocular image. $M_{\left(n,m\right)}$ is the pixel value of the monocular image at the (n,m) pixel position and $O_{\left(n,m\right)}$ is the displacement of frames in the optical flow map at the (n,m) pixel position. When the $O_{\left(n,m\right)}$ is 0, the color of optical flow heat map is deep blue as shown in Figure 8a. We do not use all optical flow pixels to estimate depth since these pixels can be also noises to prevent accurate depth estimation. Therefore we need to select optical flow pixels to use optical flow information efficiently. *i* is interval between the monocular image the pixel value and optical flow map value. In this way, the optical flow map can be used effectively.

## 5. Experiments

In this section, we evaluate our method in terms of accuracy, latency and the performance to avoid collisions.

**Figure 9:**
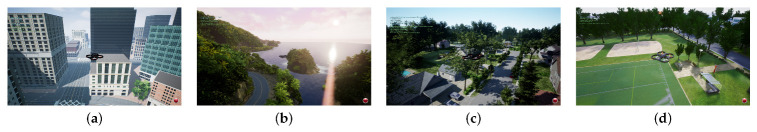
Appearance of the maps for training: (**a**) City environment, (**b**) Coastline, (**c**) Neighborhood, (**d**) Soccer field.

We use Intel Core i7-9700K (32 GB of main memory) and NVIDIA GeForce RTX 2070 SUPER, which is represented in Table 1. Dataset, which are used for training, validation, and testing, have been collected from four maps provided in the AirSim environment; Blocks, City, Coastline, and Neighborhood, where the overviews of the maps are shown in Figure 9.

**Figure 10:**
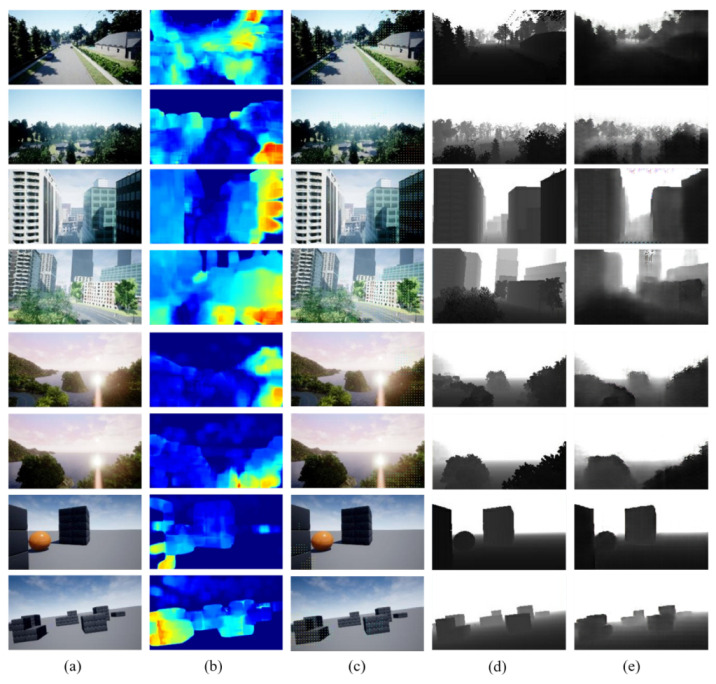
Inputs and outputs: (**a**) RGB images, (**b**) Optical flow maps, (**c**) RGB images embedded with optical flow map, (**d**) Ground truth, (**e**) Depth estimation maps.

We train our model in the following conditions: the number of epochs is set to 100. The batch size is set to 1, and the lambda of L1 norm is set to 100. In the experiments, we have prepared 16,000 pairs of monocular and depth images for each of the maps. 8000 pairs out of 16,000 are used to training our Pix2Pix-based model. The rest of the pairs in monocular and depth images is employed to test out model. In the labelling process, the depth and monocular images are taken through multiple flights with a variety of routes in AirSim beforehand. Figure 10 shows the examples of the inputs and outputs of the model trained with the parameters. Figure 10a shows the RGB images taken by a monocular camera during flights in the four maps of AirSim. At the same time, we obtain the optical flow maps as shown in Figure 10b. From the images, we derive RGB images with embedding an optical flow map in Figure 10c. Compared to the ground truth images in Figure 10d, our proposed method generates depth images as shown in Figure 10e.

### 5.1. Preliminary Evaluation with Different Pixels Interval of Optical Flow Maps

In this experiment, we use six models to investigate the effect of the optical flow maps, and the accuracy and error are compared. One out of six models employs only optical flow maps as input for depth estimation. The others embed the optical flow map into the monocular image at different intervals. The embedding intervals are one, three, five, seven, and nine pixels intervals. We intuitively suppose that the dense pixels of the optical flow map provide much information and achieve higher accuracy than the sparse pixels.

In order to quantify estimation error of models, we use rooted mean squared error (*RMSE*) and absolute relative error (*Rel*.) metrics. Hereby, *RMSE* is obtained by the following equation.

(19) $$RMSE=\sqrt{\frac{1}{N}\sum_{i=1}^{N}{\left(y_{i}^{gt}-y_{i}\right)}^{2}}$$

$y_{i}^{gt}$ is ground truth value. $y_{i}$ is estimation value. *N* is number of data. *Rel.* is obtained by the following equation.

(20) $$Rel.=\frac{1}{N}\sum_{i=1}^{N}\frac{\left|\right|y_{i}^{gt}-y_{i}\left|\right|}{y_{i}^{gt}}$$

Specifically, the accuracy metrics are defined as:

(21) $$\delta_{n}=\frac{\mathrm{Card}\left(\left\{y_{i}:max\left(\frac{y_{i}}{y_{i}^{gt}},\frac{y_{i}^{gt}}{y_{i}}\right)<1.25^{n}\right\}\right)}{\mathrm{Card}\left(\{y_{i}\}\right)}\;\left(n=1,2,3\right)$$

Table 2 shows the error and accuracy of each model.

The results show that the model with the five pixel interval remarks the lowest error and the highest accuracy. In addition, the model trained with only optical flow maps shows the highest error and lowest accuracy. In terms of *RMSE* and *Rel.*, the model trained with only optical flow maps increases 1.6736 points compared to the model with five pixels intervals. As well as the accuracy, the model with five pixels intervals achieves the highest value for each delta metric. The results imply that many pixels intervals might be about to cause over-fitting and lose information of the original RGB images, resulting in the high *RMSE* and *Rel.*, especially over seven pixels intervals. In contrast to the error metrics, the more pixels intervals achieve the improvement of the accuracy.

### 5.2. Comparison Accuracy between Proposed Method and Related Work

We evaluate our model in terms of the error and accuracy, compared to the model presented in [12]. The compared model is trained without using the optical flow. In other words, this model uses only RGB images to generate depth estimation maps. For our proposed model, we utilize the optical flow embedded into RGB images. The model is selected with five pixels intervals, which represents the lowest *RMSE* and *Rel.* in the Table 2.

Table 3 shows the results of the error and accuracy comparison. Compared to the model without optical flow, we have demonstrated that embedding the optical flow enables to achieve the slightly lower error and higher accuracy.

As shown in Table 3, in Shimada, T. et al. method, *RMSE* is 5.942, *Rel.* is 0.1338, $\delta_{1}$ is 0.8871, $\delta_{2}$ is 0.9562, $\delta_{3}$ is 0.9772. In proposed method, *RMSE* is 6.005, *Rel.* is 0.1230, $\delta_{1}$ is 0.8923, $\delta_{2}$ is 0.9608, $\delta_{3}$ is 0.9796. in Shimada, T. et al. method, *RMSE* is 5.942, *Rel.* is 0.1338, $\delta_{1}$ is 0.8871, $\delta_{2}$ is 0.9562, $\delta_{3}$ is 0.9772. In *RMSE*, Shimada, T. et al. method was better than proposed method. On the other hand, proposed method is superior in other evaluation indicators.

To confirm whether our method is effective in a real environment, we test our model using the KITTI dataset [24], as shown in Table 4. The KITTI dataset contains RGB images and depth images taken in the real world. We compare our AirSim-based model with other models trained on real images proposed by related works. Although the results of our method are slightly lower than those of association studies based on real model training, it is still a good result.

### 5.3. Run Time Evaluation

We also evaluate run time. We evaluate for the servers and the embedded devise, which represent NVIDIA RTX 2070 SUPER, Intel Core i7 9700K, and Jetson Xavier NX.

Table 5 shows the results of the run time per image. The slowest run time is shown in the Jetson and represents 0.193 s. In other words, approximately five frames per second can be processed in the Jetson. On the other hand, the result in the NVIDIA RTX 2070 SUPER shows 0.031 s per image. The validation of the results for collision avoidance depends on how long our model can estimate the distance in generated depth images.

We have concluded that the processing time is sufficient to avoid collisions in real time. NVIDIA Jetson Xavier NX is a small board computer that can be mounted on a UAV. The weight of Jetson Xavier NX is about 180 g. On the other hand, there is an accurate depth sensor Velodyne HDL-64E used in KITTI dataset [24]. The weight of HDL-64E is 12,700 g [37]. The weight of the other depth sensors which can measure 200 m are also near 1000 g. From the above, Jetson is light enough compared to long range depth sensors like used in KITTI dataset [24]. Jetson is lighter than long range depth sensor. In addition, unlike attaching such a sensor, the replacement from a base-board into Jetson Xavier NX does not increase the weight so much.

### 5.4. Collision Rate Evaluation in AirSim Environment

**Figure 11:**
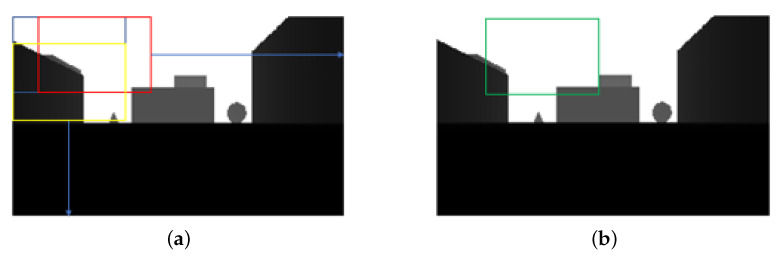
Direction decision from divided sections in [6]. (**a**) Overlapped section, (**b**) Section selection.

Previously, we have evaluated the accuracy and run time of the proposed method. In this section, we conduct the simulation of a drone flight in AirSim to demonstrate that the proposed method can fly avoiding collision with objects. In order to realize the safe flight of an autonomous drone, it is necessary to plan the path by itself, that is, the drone needs to select the direction so that the drone can avoid colliding with objects. In the experiments, we use a state-of-the-art path planning method for flight control, which is developed in [6]. The work in [6] introduced the method that divides a depth map into multiple sections. The presented method in [6] divides a depth image into 289 overlapped sections (17 rows and 17 columns) as shown in Figure 11.

By dividing into overlapped sections, the drone selects the best section to avoid obstacles and pass safely so that the drone determines the section with the maximum total pixel value. The flight is simulated 400 times in the four maps. The flight scenarios are randomly generated in terms of route, direction, and distance.

We compare the collision rates that the number of collisions account for towards the total number of flights. Hereby, we define the collision rate for a map in the following formula:

(22) $$Collision\;Rate=\frac{No.\;of\;Collisions}{No.\;of\;Flights\;(i.e.,\;400\;flights\;in\;total)}$$

Note that we assume that the flight has a collision if the drone collides with an obstacle even once during its flight.

In the experiments, we use the following four methods: The first can measure up to 10 m, which assumes a real depth camera for reasonable price and low weight enough to equipped on a drone. The second can measure up to 200 m. This method assumes an ideal depth camera, where it can measure by up to 200 m but is too heavy to be mounted on a drone in the real world. This method is used as ground truth depth images for comparison. The third is presented by Shimada, T. et al. [12]. This method inputs a monocular image to generate a depth image through Pix2Pix. The fourth is our proposed method. Our method combines an image with optical flow map into Pix2Pix, and it generates the estimated depth map.

Table 6 shows the results of the collision rate in each map of AirSim. The results show that our proposed method achieves the lower collision rate compared to the method presented in [12]. The depth map for 10 m yields the highest collision rate, and the result explicitly indicates that inaccurate depth images are useless to collision avoidance. The method [12] represents that it achieves the higher collision rate than the proposed method. The results are attributed to depth maps with the low error and high accuracy.

## 6. Discussion

### 6.1. Evaluation for Effects of Pixels Interval

**Figure 12:**
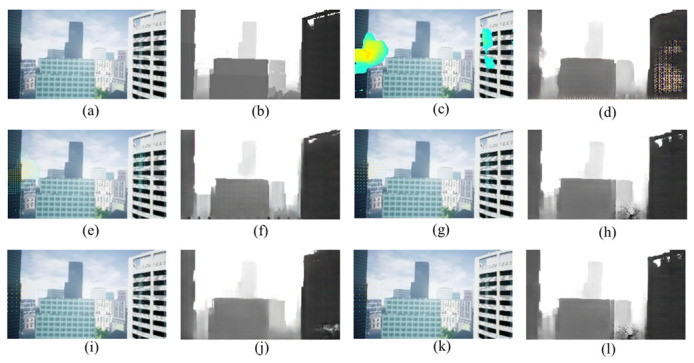
Inputs and outputs of each model: (**a**) RGB image, (**b**) Ground truth, (**c**) One pixels interval model input, (**d**) One pixels interval model output, (**e**) Three Pixels interval model input, (**f**) Three pixels interval model output, (**g**) Five pixels interval model input, (**h**) Five pixels interval model output, (**i**) Seven pixels interval model input, (**j**) Seven pixels interval model output, (**k**) Nine pixels interval model input, (**l**) Nine pixels interval model output.

We discuss that the reason why five pixels interval model achieves the highest accuracy and the lowest error. Figure 12 shows inputs and outputs each model. As shown in Figure 12, the input of the one pixel interval model is filled with optical flow of monocular image features, and the output is distorted. The input of the three pixel interval model is also filled with optical flow pixels. On the other hand, The input of the input of seven pixel interval model and the input of nine pixel model are not enough optical flow pixels. Therefore, five pixels interval model is superior to the others.

**Figure 13:**
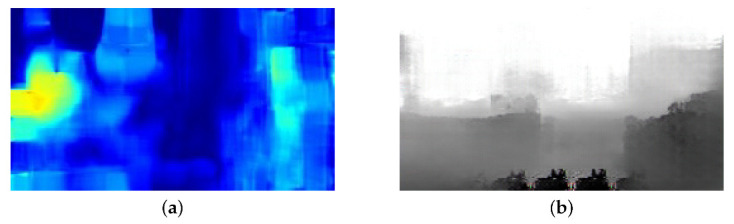
Input and Output of Optical Flow Model: (**a**) Input, (**b**) Output.

Figure 13 shows the input and output of the model which is trained using only optical flow maps. Figure 13a is generated from two adjacent frames of Figure 12a and the previous frame of it. As shown in Figure 13, the output of optical flow model deviates from the ground truth. The reason is that an optical flow map alone cannot accurately capture objects such as buildings if the Pix2Pix-based model is utilized.

### 6.2. Comparison of the Error of Depth Information

**Figure 14:**
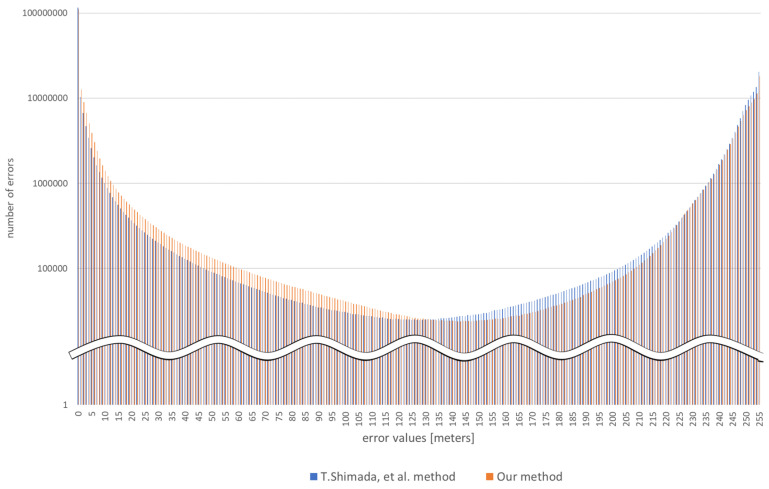
Error Distribution.

Figure 14 shows error distribution.

In this Figure 14, the horizontal axis shows the value of the error and the vertical axis shows the number of errors. The error value is in meters. The blue bars show the error distribution of Shimada, T. et al. method [12], and the orange bars show the error distribution of the proposed method. As can be seen from this Figure 14, the error of the proposed method is within a smaller range than that of Shimada, T. et al. method [12]. Therefore, it is believed that the proposed method was superior in terms of accuracy and error. In addition, the proposed method has fewer outliers, so the collision rate is considered to be lower than that of Shimada, T. et al. method.

In addition, according to Table 4, although proposed method is higher *RMSE* than other methods, the proposed method is higher accuracy. The reason for this is that the proposed method embeds optical flow pixels in the monocular image, which increases the outliers in those pixels, but improves the accuracy of the surrounding pixels. Therefore, while *RMSE* is degraded due to outliers at that one point, the overall accuracy is high and the value of $\delta_{n}$ is better than other methods. It can be seen from the Table 6 that the outlier at this single point is not a problem for drone collision avoidance.

## 7. Conclusions

This paper presents the use of Pix2Pix with optical flow to obtain highly accurate depth maps to avoid drone collisions. We have developed an effective way to embed optical flow diagrams in depth estimation. The collision rate of the proposed method is lower than a state of work, over-performing the related works. Even though we used an old image generation method called Pix2Pix, we were able to improve the accuracy of depth estimation by devising a new input image. In addition, we were able to adapt the model trained in the virtual environment to the real world and obtain results comparable to other methods. Even when Pix2Pix with optical flow is used, the results showed that there were few collisions. In order to implement the system on a real drone, it is necessary to install a high-performance computer. Our future work is to study and experiment on how to increase the speed of the system so that it can be used in actual drones. The investigation of generalization performance is also a future task. In addition, we will improve the method more effectively embeds an optical flow map into a monocular image. Finally, we will experiment with real drones and quantitatively evaluate the effectiveness of the proposed method in a real environment.

## Figures and Tables

**Table 1:** Experimental Environment.

OS	Windows 10 pro
RAM	32 GB 2666 MHz
CPU	Intel Core i7-9700K 3.60 GHz
GPU	NVIDIA GeForce RTX 2070 SUPER 8 GB

**Table 2:** Preliminary evaluation of our proposed method regarding the error and accuracy.

Pixel Interval	Error (Lower Is Better)		Accuracy (Higher Is Better)		
	*RMSE*	*Rel.*	$\delta_{1}$	$\delta_{2}$	$\delta_{3}$
Optical flow only	7.6786	0.3886	0.7416	0.8604	0.9129
1 pixel	6.6258	0.1675	0.8634	0.9376	0.9621
3 pixels	6.0947	0.1397	0.8878	0.9554	0.9758
5 pixels	6.0050	0.1230	0.8923	0.9608	0.9797
7 pixels	6.5064	0.1240	0.8910	0.9605	0.9795
9 pixels	6.7068	0.1335	0.8947	0.9573	0.9762

**Table 3:** Comparison to the state-of-the-art method using AirSim dataset.

Method	Error (Lower Is Better)		Accuracy (Higher Is Better)		
	*RMSE*	*Rel.*	$\delta_{1}$	$\delta_{2}$	$\delta_{3}$
Shimada [12]	5.942	0.1338	0.8871	0.9562	0.9772
Proposed method	6.005	0.1230	0.8923	0.9608	0.9796

**Table 4:** Comparison to the other methods using KITTI dataset.

Method	Error (Lower Is Better)		Accuracy (Higher Is Better)		
	*RMSE*	*Rel.*	$\delta_{1}$	$\delta_{2}$	$\delta_{3}$
Eigen et al. [34]	7.156	1.515	0.692	0.899	0.967
Liu et al. [35]	6.986	0.217	0.647	0.882	0.961
Kuznietsov et al. [36]	4.621	0.113	0.862	0.960	0.986
Proposed method	7.605	0.154	0.813	0.958	0.985

**Table 5:** Runtime to generate an image.

Device	Runtime (s)	
	Non Optical Flow	Optical Flow
NVIDIA RTX 2070 SUPER	0.031	0.134
Intel Core i7 9700K	0.181	0.273
Jetson Xavier NX	0.193	0.297

**Table 6:** Comparison of collision rate.

Map	Collision Rate (%)			
	10 m	200 m	Shimada [12]	Our Method
Blocks	58.75	7.000	17.50	14.50
City environment	73.50	26.00	34.75	34.00
Coastline	70.50	0.250	1.500	1.250
Neighborhood	82.00	7.000	2.500	1.000

## Data Availability

Not applicable.
